# Protective Effect of High Protein and Calcium Intake on the Risk of Hip Fracture in the Framingham Offspring Cohort

**DOI:** 10.1002/jbmr.194

**Published:** 2010-07-26

**Authors:** Shivani Sahni, L Adrienne Cupples, Robert R Mclean, Katherine L Tucker, Kerry E Broe, Douglas P Kiel, Marian T Hannan

**Affiliations:** 1 Institute for Aging Research, Hebrew SeniorLife and Harvard Medical School Boston, MA, USA; 2 Department of Biostatistics, Boston University School of Public Health and the Framingham Heart Study Boston, MA, USA; 3 Department of Health Sciences, Northeastern University School of Health Professionals Boston, MA, USA

**Keywords:** NUTRITION, PROTEIN INTAKE, HIP FRACTURE, CALCIUM, COHORT STUDY

## Abstract

The effect of protein on bone is controversial, and calcium intake may modify protein's effect on bone. We evaluated associations of energy-adjusted tertiles of protein intake (ie, total, animal, plant, animal/plant ratio) with incident hip fracture and whether total calcium intake modified these associations in the Framingham Offspring Study. A total of 1752 men and 1972 women completed a baseline food frequency questionnaire (1991–1995 or 1995–1998) and were followed for hip fracture until 2005. Hazard ratios (HRs) were estimated using Cox proportional hazards regression adjusting for confounders. Baseline mean age was 55 years (SD 9.9 years, range 26 to 86 years). Forty-four hip fractures occurred over 12 years of follow-up. Owing to significant interaction between protein (total, animal, animal/plant ratio) and calcium intake (*p* interaction range = .03 to .04), stratified results are presented. Among those with calcium intakes less than 800 mg/day, the highest tertile (T3) of animal protein intake had 2.8 times the risk of hip fracture [HR = 2.84, 95% confidence interval (CI) 1.20–6.74, *p* = .02] versus the lowest tertile (T1, *p* trend = .02). In the 800 mg/day or more group, T3 of animal protein had an 85% reduced hip fracture risk (HR = 0.15, 95% CI 0.02–0.92, *p* = .04) versus T1 *(p* trend = .04). Total protein intake and the animal/plant ratio were not significantly associated with hip fracture (*p* range = .12 to .65). Our results from middle-aged men and women show that higher animal protein intake coupled with calcium intake of 800 mg/day or more may protect against hip fracture, whereas the effect appears reversed for those with lower calcium intake. Calcium intake modifies the association of protein intake and the risk of hip fracture in this cohort and may explain the lack of concordance seen in previous studies. © 2010 American Society for Bone and Mineral Research.

## Introduction

The prevalence of osteoporosis in the United States is estimated to increase (based on 2000 Census data) from approximately 10 million to over 14 million people in 2020.(1) In 2005, more than 2 million incident osteoporotic fractures were estimated to have occurred in the United States, with direct medical costs of approximately $17 billion.(2) Hip fractures are the most serious type of fractures because they almost always result in hospitalization, lead to permanent disability in about 50% of patients, and are fatal in approximately 20% of patients.(3)

Previous studies of protein intake and a variety of skeletal outcomes have been conflicting possibly because of a variety of factors, including the level of protein in the diet, the protein source, calcium intake, weight loss, and acid-base balance of the diet.(4) Small, short-duration metabolic balance studies have established that increasing dietary protein elevates urinary calcium excretion and creates negative calcium balance.(5–7) However, these metabolic studies may not accurately reflect the overall effects of protein on the skeleton because evidence from some,(8–14) but not all,(15–19) population-based studies has shown that protein intake is beneficial for the skeleton. Furthermore, the influence of protein on bone health may differ based on calcium intake. A randomized, placebo-controlled trial of elderly men and women concluded that higher protein intake may protect against bone loss only in individuals supplemented with calcium citrate and vitamin D.(20) A cross-sectional study from the National Health and Nutrition Examination Survey (NHANES) reported that in postmenopausal women (aged ≥ 50 years) who consumed less than 46 g/day of dietary protein, those with a total calcium intake of 1200 mg/day or more had a significantly higher risk of fracture than those with the lowest total calcium intake, whereas in women who consumed more than 70 g/day of dietary protein, those with a total calcium intake of 1200 mg/day or more had an insignificant lower risk of fracture.(21) To our knowledge, no previous study has examined whether the association of protein intake with the risk of hip fracture is modified by total calcium intake at the levels typically consumed in the United States. Therefore, we hypothesized that higher intake of protein (ie, total, plant, animal protein, and animal/plant protein ratio) would be associated with a reduced risk of hip fracture in middle-aged and older adults participating in the Framingham Offspring Cohort. Furthermore, we hypothesized that individuals with higher protein intake would have lower risk of hip fracture when calcium intake is high.

## Methods

### Participants

In 1971, the Framingham Offspring Study was initiated by enrolling 5124 adult children of the (original) Framingham Study cohort and their spouses. The purpose of this study was to identify risk factors in the etiology of coronary artery disease, including familial factors.(22) Every 4 to 8 years, offspring participants have had physical examinations, blood chemistries, assessment of risk factors, and questionnaires. Of the 5124 originally enrolled Framingham Offspring Cohort participants, 1752 men and 1972 women completed a food frequency questionnaire (FFQ) in either 1991–1995 or 1995–1998 (baseline exam for this study) and were followed for hip fracture until 2005. We excluded participants with missing/incomplete FFQs (based on the criteria of more than 12 food items left blank on the FFQ) or with energy intakes of less than 2.51 or more than 16.74 MJ (<600 or >4000 kcal/day) at the baseline exam (*n* = 59). We further excluded 68 participants owing to missing covariate information on weight, height, physical activity index, menopause status, or smoking status and another 13 subjects who fractured prior to the baseline exam. Therefore, the final analytic sample included 3656 Framingham Offspring Cohort study members. All participants provided informed consent for their participation. This study was approved by the Institutional Review Boards at Boston University and Hebrew SeniorLife.

### Assessment of dietary protein intake

Usual dietary intake was assessed with the semiquantitative 126-item Willett FFQ at the baseline exam for this study (in either 1991–1995 or 1995–1998).(23,24) This FFQ has been validated against multiple diet records and blood measures for many nutrients, including protein, in several populations.(25,26) Questionnaires were mailed to the study participants prior to their scheduled clinic visit. They were asked to complete them, based on their intake over the previous year, and to bring them to the clinic examination, where they were reviewed with participants by clinic staff. Intakes of total protein (g/day), plant protein (g/day), and animal protein (g/day) were assessed using the food list section of the FFQ. We calculated the animal/plant protein intake ratio.

### Assessment of fracture

Using the assessment protocol that has been reported previously,(27) hip fractures were reported by interview at each examination (conducted every 4 years) or by telephone interview for participants unable to attend examinations. All except three reported hip fractures were confirmed by review of medical records and radiographic and operative reports. An incident hip fracture was defined as a first-time fracture of the proximal femur. Study participants were followed for hip fracture from the date of the dietary assessment through December 2005.

### Potential confounding factors

Age (years), height (m), weight (kg), smoking (current versus noncurrent), physical activity index, and menopause status (yes/no) in women, dietary calcium (mg/day), total vitamin D (IU/day), total energy (MJ/day), and calcium supplement use (yes/no) were measured at the baseline exam for this study (in either 1991–1995 or 1995–1998). The dietary intakes and supplement use were assessed using the FFQ. Intakes of total calcium, dietary calcium, total vitamin D (from diet + supplements), total energy, and calcium supplement use were measured using the food list section of the FFQ. Height was measured without shoes, in inches, and weight was recorded in pounds with a standardized balance-beam scale. Smoking status, assessed by questionnaire, was used to classify individuals as either current smokers or former/never smokers. Physical activity was measured with the Framingham physical activity index, which asked about number of hours spent in heavy, moderate, light, or sedentary activity and number of hours spent sleeping during a typical day.(28) The physical activity index at the 1989–1992 exam was used for subjects who were missing the physical activity index at the 1995–1998 exam. For those whose physical activity index remained missing, we used an average of physical activity from the 1983–1986 exam and the 1998–2001 exam.

### Statistical analysis

Dietary protein, animal protein, plant protein, and dietary calcium intakes were normally distributed. All protein exposures were adjusted for total energy intake using the residual method.(24) As per this method, protein intakes were regressed on total energy intake to create residuals. Protein intake residuals then were added to a constant, where the constant equals the predicted nutrient intake for the mean energy intake of the study population. Protein intake was modeled as both a continuous variable and using tertiles. We used Cox proportional hazards regression to calculate the hazard ratio (HR) and 95% confidence interval (CI) estimating the relative increase in the risk of hip fracture for each 1 unit increase in each of the protein intake variables and for the upper 2 tertiles of protein intake versus the lowest tertile. We also tested for a linear trend across tertiles. Crude incidence rates in each tertile of the protein exposure also were calculated. Furthermore, we tested for interaction with total calcium intake (<800 mg/day versus ≥800 mg/day, the median intake of total calcium in this cohort) by including an interaction term in the regression model. If a significant interaction was observed (*p* < .05), regression models were repeated stratified by total calcium intake group. The final models were adjusted for dietary calcium intakes within each stratum.

Models were adjusted for sex, age, weight, height, total energy intake, physical activity index, smoking, menopause status, and intake of dietary calcium, calcium supplements, and total vitamin D at the baseline exam. Models with plant and animal protein intakes as the independent variables were adjusted for each other, and models for animal/plant protein ratio were adjusted for total protein intake. Analyses were conducted with both men and women combined and separately. For analyses on the combined sample of men and women, we created an indicator variable to adjust for sex and menopause status (yes/no) simultaneously (group 1: men; group 2: premenopausal women; group 3: postmenopausal women). The final models within each strata of total calcium intake were further adjusted for dietary calcium intake to account for any residual confounding.

All analyses were performed using SAS statistical software (SAS Institute, Inc., SAS User's Guide, Version 9.1, Cary, NC, USA). A nominal two-sided *p* value of less than .05 was considered statistically significant for all the analyses.

## Results

### Participant characteristics

Women represented half (53%) the study sample. The mean age of men and women was approximately 55 years, and mean weight was 87 kg for men and 70 kg for women (Table 1). One-fifth of the men and women currently smoked cigarettes. More women than men (29% versus 13%) reported calcium supplement use. The mean intake of dietary calcium was 751 mg/day in men and 739 mg/day in women, whereas that of total calcium (diet + supplements) was 776 and 872 mg/day, respectively. The mean protein intake was 79 g/day in men and 76 g/day in women. Over the 12 years of follow-up, 44 incident hip fractures were reported among 3656 participants. Incidence rates for hip fracture were 1.2 per 1000 person-years for the lowest tertile of total protein intake, 0.98 per 1000 person-years for the second tertile and 0.90 per 1000 person-years for the highest tertile of total protein intake [incidence rate ratio (IRR) of T2 versus T1 = 0.82, 95% CI 0.40–1.67, *p* = .59; IRR of T3 versus T1 = 0.76, 95% CI 0.37–1.57, *p* = .46, *p* trend = .46].

**Table 1:** Characteristics of the Study Participants of the Framingham Offspring Cohort at the 1991–1995 or 1995–1998 Baseline Examination

Descriptive variables	Men (*n* = 1725)	Women (*n* = 1931)
Age (years)	55.3 ± 9.9^a^	54.9 ± 9.8
Weight (kg)	87 ± 14.3	70 ± 14.8
Height (m)	1.8 ± 0.07	1.6 ± 0.06
Body mass index (BMI; kg/m^2^)	28.1 ± 4.1	26.8 ± 5.5
Physical activity index	37.5 ± 7.8	36.6 ± 6.0
Current smokers (%)	18.6	19.3
Calcium supplement use (%)	13.0	29.2
Postmenopausal women (%)	—	68.9
Hip fracture (*n*)	10	34
Intake of		
Total energy (MJ/day)	8.2 ± 2.6	7.3 ± 2.4
Total calcium (mg/day)^b^	776 ± 381	872 ± 472
Dietary calcium (mg/day)	751± 366	739 ± 358
Total vitamin D (IU/day)^b^	294 ± 235	318 ± 256
Dietary vitamin D (IU/day)	204 ± 132^a^	197 ± 129
Total protein (g/day)	79.0 ± 27	75.7 ± 27
Animal protein (g/day)	54.3 ± 22	52.5 ± 22
Plant protein (g/day)	24.6 ± 9	23.1 ± 9
Animal/plant protein ratio	2.4 ± 1	2.4 ± 1

a Mean ± SD.

b Total nutrient intake = dietary intake + supplemental intake.

### Association between protein and hip fractures

In the tertile analysis, individuals in the highest tertile of animal protein intake (median 68 g/day) had an increased risk of hip fracture compared with subjects in the lowest tertile of animal protein intake (median 38 g/day) (T3 HR = 2.08, 95% CI 0.97–4.47, *p* = .06; T2 HR = 1.40, 95% CI 0.66–3.00, *p* = .38, *p* trend = .05). However, the trend was only marginally significant. Similar associations also were observed for the ratio of animal to plant protein intake (T3 HR = 2.14, 95% CI: 0.93-4.93, *p* = 0.07; T2 HR = 0.93, 95% CI 0.42–2.08, *p* = .87, *p* trend = .09). In contrast, while not statistically significant, individuals in the highest tertile of plant protein intake (median 29 g/day) tended to have fewer hip fractures than subjects in the lowest tertile of plant protein intake (median 18 g/day) (T3 HR = 0.48, 95% CI 0.20–1.14, *p* = .10; T2 HR = 0.96, 95% CI 0.48–1.92, *p* = .91, *p* trend = .10). No significant associations were observed for other protein exposures and risk of hip fracture (*p* trend ranged from .27 to .52; data not shown).

### Interaction by total calcium intake

Statistically significant interactions were observed for protein exposures and total calcium intake in the combined sample of men and women (*p* for interaction = .04 for total protein intake, .03 for animal protein intake, and .04 for animal/plant protein ratio; data not shown**)**. The analyses then were stratified by total calcium intake (<800 mg/day versus ≥800 mg/day).

### Low-calcium group (total calcium intake < 800 mg/day)

In the continuous analyses, participants with a higher animal/plant protein ratio tended to have an increased risk of hip fracture (HR = 1.38, 95% CI 0.98–1.94, *p* = .07). No significant associations were observed for other protein exposures and the risk of hip fracture (*p* trend ranged from .17 to .44; data not shown).

In the tertile analysis, individuals in the highest tertile of animal protein intake (median 60 g/day) had a significantly higher risk for hip fractures than subjects in the lowest tertile of animal protein intake (median 34 g/day) (T3 HR = 2.84, 95% CI 1.20–6.74, *p* = .02; T2 HR = 0.94, 95% CI 0.32–2.69, *p* = .91, *p* trend = 0.02) (Table 2). These associations remained significant after adjustment for dietary calcium intake (T3 HR = 3.18, 95% CI 1.30–7.77, *p* = .01; T2 HR = 0.97, 95% CI 0.33–2.78, *p* = .96, *p* trend = .01). No significant associations were observed for other protein exposures and risk of hip fracture (*p* trend ranged from .12 to .26) in this subgroup with total calcium intake of less than 800 mg/day.

**Table 2:** Association of Protein Intake With the Risk of Hip Fracture in Men and Women From the Framingham Offspring Cohort

	Hazard ratio^b^ for tertiles of protein intake							
	
	Total calcium intake < 800 mg/day,^c^ *n* = 2124, *n* events = 29				Total calcium intake ≥ 800 mg/day,^c^ *n* = 1532, *n* events = 15			
		
Protein exposures^a^	T1 ref)	T2	T3	*p* trend	T1 (ref)	T2	T3	*p* trend
Total protein (g/day), *n* events	10	9	10	—	7	5	3	—
Total protein (g/day)	1.0	1.41 (0.56–3.56)	2.02 (0.83–4.94)	.12	1.0	0.66 (0.20–2.20)	0.30 (0.07–1.25)	.09
Total protein (g/day)^d^	1.0	1.46 (0.58–3.70)	2.20 (0.88–5.54)	.09	1.0	0.70 (0.20–2.40)	0.54 (0.12–1.30)	.38
Animal protein (g/day), *n* events	9	6	14		5	8	2	—
Animal protein (g/day)^e^	1.0^a^	0.94 (0.32–2.69)^a^ ^b^	2.84 (1.20–6.74)^b^	.02	1.0^a^	1.16 (0.33–3.90)^a^ ^b^	0.15 (0.02–0.92)^b^	.04
Animal protein (g/day)^d^ ^e^	1.0^a^	0.97 (0.33–2.78)^a^ ^b^	3.17 (1.30–7.78)^b^	.01	1.0	1.51 (0.43–5.31)	0.32 (0.05–2.08)	.33
Plant protein (g/day), *n* events	11	13	5		6	6	3	
Plant protein (g/day)^e^	1.0	1.02 (0.43–2.40)	0.56 (0.19–1.68)	.28	1.0	0.77 (0.23–2.59)	0.24 (0.06–1.06)	.07
Plant protein (g/day)^d^ ^e^	1.0	1.10 (0.46–2.64)	0.60 (0.20–1.85)	.34	1.0	0.59 (0.17–2.04)	0.23 (0.05-1.03)	.06
Animal/plant protein, *n* events	11	4	14		5	7	3	
Animal/plant protein^f^	1.0	0.46 (0.14–1.48)	1.86 (0.69–4.99)	.26	1.0	2.24 (0.66–7.56)	1.20 (0.23–6.21)	.65
Animal/plant protein^d^ ^f^	1.0	0.45 (0.14–1.45)	1.81 (0.68–4.86)	.29	1.0	2.50 (0.70–8.87)	2.02 (0.37-11.05)	.32

a Multivariate models adjusted for sex and menopause status (group 1: men; group 2: premenopausal women; group 3: postmenopausal women), age (years), weight at baseline (kg), height at baseline (m), physical activity index, intake of energy (MJ/day) and total vitamin D (IU/day), and smoking status (current versus former/never). Protein exposures were energy-adjusted residuals added to a constant, where the constant equals the nutrient intake for the mean energy intake of the study population.

b Hazard ratio (HR) with different superscripts are significantly different from HR of tertile 1 at *p* < .05.

c Range (median intake of dietary calcium) in subjects with <800 mg/day of calcium intake by tertiles of total protein intake (g/day): T1 = 109–799 (517) mg/day; T2 = 108–799 (525) mg/day; and T3 = 146–799 (578) mg/day. Range (median intake of dietary calcium) in subjects with ≥800 mg/day of calcium intake by tertiles of total protein intake (g/day): T1 = 188–2550 (920) mg/day; T2 = 199–2056 (950) mg/day; and T3 = 206–3283 (1096) mg/day.

d Models were additionally adjusted for dietary calcium intake.

e HRs for animal and plant protein intakes were estimated from the same model, adjusting for each other.

f Models for animal/plant protein ratio were additionally adjusted for total protein intake.

### High-calcium group (total calcium intake ≥ 800 mg/day)

In the continuous analyses, protective effects of greater protein intake were observed for total protein (HR = 0.95, 95% CI 0.92–0.99, *p* = .008) and animal protein intake (HR = 0.95, 95% CI 0.92–0.99, *p* = .009; data not shown). These associations remained marginally significant after further adjustment for dietary calcium intake (for total protein intake, HR = 0.97, 95% CI 0.92–1.00, *p* = .07, and for animal protein intake, HR = 0.97, 95% CI 0.93–1.00, *p* = .08).

In the tertile analyses, participants in the highest tertile of total protein intake (median 103 g/day) tended to have a decreased risk of hip fracture relative to subjects in the lowest tertile of total protein intake (median 79 g/day) (T3 HR = 0.30, 95% CI 0.07–1.25, *p* = .09; T2 HR = 0.66, 95% CI 0.20–2.20, *p* = .50, *p* trend = .09) (Table 2). These associations did not reach statistical significance, and the marginal trend lost significance after further adjustment for dietary calcium intake (*p* trend = .38). Similarly, subjects in the highest tertile of animal protein intake (median 76 g/day) had a significantly lower risk of hip fractures than subjects in the lowest tertile of animal protein intake (median 48 g/day) (T3 HR = 0.16, 95% CI 0.02–0.92, *p* = .04; T2 HR = 1.15, 95% CI 0.33–3.90, *p* = .82, *p* trend = .04) (Table 2**)**. This association also lost significance after further adjustment for dietary calcium intake (*p* trend = .33). Similarly, subjects in the highest tertile of plant protein intake (median 34 g/day) tended to have a lower risk of hip fractures than subjects in the lowest tertile of plant protein intake (median 22 g/day) (T3 HR = 0.24, 95% CI 0.06–1.06, *p* = .06; T2 HR = 0.77, 95% CI 0.23–2.59, *p* = .68, *p* trend = .07) (Table 2). No significant associations were observed for animal/plant protein ratio and the risk of hip fracture (*p* trend = .65).

## Discussion

In this study we found that calcium intake modified the association between protein intake and hip fracture risk in our cohort of middle-aged and older adults over 12 years of follow-up. Among those with calcium intakes of less than 800 mg/day, the highest tertile of animal protein intake had 2.8 times the risk of hip fracture versus the lowest tertile (HR = 2.84, 95% CI 1.20–6.74). In the 800 mg/day or more of calcium group, the highest tertile of animal protein had an 85% *reduced* hip fracture risk versus the lowest tertile (HR = 0.15, 95% CI 0.02–0.92). Total protein and plant protein intake also showed borderline protective effects (Table 2; *p* range = .07 to .09) in the high-calcium-intake group.

Most population-based observational studies suggest that greater dietary protein intake is associated with higher bone mineral density (BMD) values in middle-aged and older adults.(8,10–14,29) However, relatively few observational studies have examined the association of protein intake with the risk of fracture.(9,21,30–32) Results of previous studies of protein and fracture have yielded conflicting results. As pointed out by Heaney and Layman in a recent review of this topic, the effect of protein on bone can vary with a variety of factors, including the level of protein in the diet, the protein source, calcium intake, weight loss, and acid-base balance of the diet.(4)

### Interaction of protein intake with calcium

Results from this study are in agreement with the work conducted by Dawson-Hughes and colleagues suggesting that the positive effects of dietary protein on bone may be realized only in the setting of adequate calcium intake. Dawson-Hughes and colleagues examined protein intake in interaction with calcium supplementation using data from a longitudinal calcium supplementation trial.(20) They reported that higher protein intake was protective of BMD loss over the 3-year follow-up, but only among the group taking calcium and vitamin D supplements. They suggested that greater absorbed calcium in the supplemental group might have offset potential negative effects of protein on calcium balance, thereby allowing positive effects of protein on the skeleton. However, there was no benefit from supplementation among those with lower intakes of protein. Similar results also were reported for fractures by E3N (Etude Epidémiologique de femmes de la Mutuelle Générale de l'Education Nationale), which is a prospective study among members of the Mutuelle Générale de l'Education Nationale (MGEN) and includes French postmenopausal women (*n* = 36,217). That study reported that high acid-ash diets were associated with an increased risk of fracture when calcium intake was low (<400 mg/1000 kcal) [relative risk (RR) =1.5 for highest versus lowest quartile, 95% CI 1.17–1.94].(33) A recent cross-sectional study using the data from the NHANES reported that in postmenopausal women (aged ≥ 50 years) who consumed less than 46 g/day of dietary protein, those with a total calcium intake of 1200 mg/day or more had a significantly higher risk of fracture than those with the lowest total calcium intake [adjusted odds ratio (OR) = 5.98, 95% CI 1.15–31.13], whereas in women who consumed more than 70 g/day of dietary protein, those with a total calcium intake of 1200 mg/day or more had an insignificant lower risk of fracture (adjusted OR = 0.69, 95% CI 0.20 –2.39).(21) In contrast, however, a recent observational study of 136 postmenopausal women noted protective effects of protein intake that were greatest when calcium intakes were less than 750 mg/day.(34) Thus the relation between protein intake and bone health may vary differently in relation to calcium intake in older adults.

### Protein source

It has been suggested that the effect of protein intake on bone metabolism varies depending on the protein source. For example, animal protein–based diets might have a greater negative effect on skeletal health than vegetable protein–based diets(30) because dietary animal protein induces a greater increase in urinary calcium excretion than vegetable protein. However, previous work by our group in the Framingham Osteoporosis Study (391 women and 224 elderly men) showed that a higher intake of animal protein was not associated with a decrease in BMD.(8) In a 3-year clinical study of 342 elderly men and women, those who consumed the most protein and were supplemented with calcium experienced the greatest improvement in BMD, and most of the protein consumed was animal protein.(20) On the other hand, some clinical studies neither support the idea that animal protein has a detrimental effect on bone health nor find that vegetable proteins are better for bone.(35,36) Our study suggests that the effect of animal protein on hip fractures was modified by total calcium intake. In our study of middle-aged men and women, persons with a greater intake of animal protein had a greater risk of hip fractures than those with lower intakes only if they had lower total calcium intakes of less than 800 mg/day. However, the hazard ratios within this group were not linear, which could be because of a threshold effect above or below a certain level or may indicate insufficient statistical power to detect a linear trend across the tertiles. In the higher calcium intake group, animal protein was protective. A similar protective trend was observed for plant protein intake, but only in the group with high calcium intake. Although it is not possible to completely isolate the protective effect of high animal protein intake from that of high calcium intake in this group because some of the animal protein comes from dairy products, most of the animal protein in this cohort came from nondairy animal sources (∼67% of animal protein intake), supporting the protective effect of animal protein intake in the high-calcium group. No significant associations were observed for plant protein intake in the low-calcium group or for animal/vegetable protein ratio in either group. This could be so because approximately 64% of total protein intake in the study subjects came from animal food sources, whereas only approximately 36% of total protein intake came from plant food sources. Low variation in the plant protein intake could have contributed to the lack of association with the risk of hip fracture. It is important to note that the mean total protein intake in this cohort was at the level of the Recommended Dietary Allowance for protein intake for this age group.

One reason that previous studies may be conflicting is that protein intake affects bone in multiple ways: (1) It contributes to the structural matrix of bone, (2) it optimizes insulin-like growth factor 1 (IGF-1), which regulates osteoblast function to help maintain bone mass, (3) it is reported to increase urinary calcium, (4) it is reported to increase intestinal calcium absorption, and (5) it may act indirectly through preservation of muscle, which itself is associated with weakness, greater risk of falls and fractures, and disability.(14,37,38) The cause of age-related muscle loss is multifactorial, and inadequate dietary protein intake may accelerate this process.(39) Thus, in different populations, the cumulative contributions of these various pathways may not be uniform.

This study is unique in that it used longitudinal, prospective data from a population-based cohort of middle-aged and older individuals followed for up to 12 years, which helps in examining causality. The Framingham Study also has collected most of the covariables and risk factors of interest for our analyses. However, this study has limitations. First, the number of hip fractures was modest, limiting the power of the study. Thus some of our *p* values did not attain traditional levels of statistical significance, although borderline statistical significance was noted and exact *p* values were stated to allow the reader to draw his or her own conclusions. At present, there is no “gold standard” tool for measuring diet. While a food diary may be better theoretically, experience shows that it may lead to underreporting, large losses in data through noncompliance, and bias owing to selected subject retention. In this study we used the FFQ to estimate dietary intakes and do not have direct measurement of grams of protein intake. However, many FFQ validity studies have shown that FFQs can rank subjects well in large epidemiologic studies.(40) Furthermore, the complete dietary data were available only at the baseline, and therefore, we were unable to adjust for any possible secular changes in diet over follow-up. In any observational study, residual confounding may occur despite our attempts to control for several potential confounders. Lastly, the results of this study are generalizable primarily to white men and women.

In conclusion, our results suggest that among middle-aged and older adults, increased animal protein intake may protect against hip fracture among those with total calcium intake of 800 mg/day or more yet may increase the risk of hip fracture for those with lower calcium intake. More studies are needed to examine these associations in larger samples with greater statistical power.
